# Imidacloprid induces hepatorenal toxicity in male albino rats via oxidative, immune inflammatory, and proliferative effects: a 90-day study

**DOI:** 10.1038/s41598-026-48767-x

**Published:** 2026-05-19

**Authors:** Soad A. Khwanes, Rania A. Mohamed, Heba Ali Abd El-Rahman, Khairy A. Ibrahim

**Affiliations:** 1 Mammalian Toxicology Department, Central Agricultural Pesticides Laboratory, Agricultural Research Center, Dokki, Giza, 12618 Egypt; 2 Department of Zoology, Faculty of Science, Cairo University, PO Box 12613, Giza, Egypt

**Keywords:** Imidacloprid, Hepatorenal, Male albino rats, Proinflammatory marker, Cellular proliferation, Biochemistry, Drug discovery, Immunology, Physiology

## Abstract

Imidacloprid (IM), a systemic neonicotinoid pesticide, is widely used globally due to its high effectiveness against a broad range of insects at minimal application rates. The current study aimed to examine how this insecticide can induce oxidative stress, disrupt the inflammatory process, and influence cellular proliferation in hepatorenal tissues, even at low doses. Thirty adult male albino rats were divided into five groups (*n* = 6). The first group served as the control, while the other four groups received IM at 0.3, 3, 30, and 60 ppm, respectively, over 90 days through drinking water. Results revealed significant histopathological changes in the liver and kidneys of rats exposed at all doses. A notable increase in hepatorenal markers, including alanine aminotransferase (ALT), aspartate aminotransferase (AST), alkaline phosphatase (ALP), urea, and creatinine levels, and a slight decrease in total serum protein (TP) were observed. There was a significant rise in malondialdehyde (MDA) levels, along with increased production of pro-inflammatory cytokines, including interleukin-6 (IL-6), tumor necrosis factor-alpha (TNF-α), and NOD-like receptor protein-3 (NLRP3). This was accompanied by strong immunopositivity of proliferating cell nuclear antigen (PCNA) and Ki-67 protein expression, while adipocyte-specific adhesion molecule (ASAM) levels showed insignificant changes. The study concludes that IM can induce hepatorenal damage accompanied by lipid peroxidation, elevated inflammatory mediators, and altered proliferative indicators. The effects were generally more apparent at higher dosages.

## Introduction

Pesticides are often used to control pests; however, excessive use raises concerns about potential health risks to humans and livestock^1^. The liver and kidneys are particularly vulnerable to this risk^2^ because of their vital roles in metabolizing and removing these chemicals from the body^3^. As a result, hepatorenal disorders have become a significant health concern^4^ related to pesticide exposure.

Neonicotinoids are popular pesticides due to their minimal soil buildup and effectiveness in managing various insect populations on plants, even at low concentrations^5^. However, they significantly pollute the environment, as seeds absorb them and later release them through leaks, drainage, and runoff^6^.

Neonicotinoid, IM, introduced in 1991, was the first neonicotinoid and became a leading global pesticide^7^. It is commonly used to protect various crops such as vegetables, fruits, and grains from pests^5^. Despite its many benefits for agriculture, IM has been shown to pose serious environmental and public health risks^8^. Additionally, it has caused the rapid decline or even extinction of several pollinators, thereby posing a significant threat to ecological balance^9^.

The United States Environmental Protection Agency^10^ has classified IM as a group E carcinogen, indicating that there is no evidence of its carcinogenicity in humans^11^. In mammals, IM is rapidly absorbed following ingestion, and the majority is metabolized in the liver, with just 16% of the initial dose excreted unchanged^12^. This decomposition occurs via two primary mechanisms: oxidative cleavage and hydroxylation^9^.

The toxicity of IM in humans can vary significantly among individuals due to differences in their cytochrome P450 isoenzymes, which mediate its oxidative metabolism. The core cause of IM’s toxicity is its ability to bind to nicotinic acetylcholine receptors^13^. This binding can disrupt nerve signals in the central nervous system of insects, leading to paralysis and ultimately death^14^. Additionally, it can damage several organs, causing numerous adverse effects^15^. These include mutagenicity^16^, teratogenic effects resulting in developmental abnormalities^17^, contributions to male infertility, and hepatorenal toxicity^18^.

A possible reason for IM toxicity is the overproduction of reactive oxygen species (ROS)^19,20^, which interferes with the body’s endogenous detoxification system^21^. These ROS can adversely affect vital cellular constituents, resulting in damage to lipids, proteins, and DNA^22^. Ultimately, this cellular damage can modify cellular structure and function^23^.

It is well established that both inflammation and oxidative stress are interconnected phenomena, as one can promote the other, leading to a toxic feedback loop that contributes to inflammation^24^. In this context, immune cells utilize proteins known as cytokines by interacting with one another and with other cell types to initiate and execute immune responses. For instance, IL-6 initiates the synthesis of acute phase proteins, which assist in modulating inflammation^25^. Moreover, TNF-α is a complex cytokine integral to inflammatory reactions^26^. In addition to these inflammatory mediators, the NLRP3 inflammasome, a crucial multiprotein complex located in the cytoplasm of mammalian cells, is influenced by several inflammatory signals^27^. This complex directly induces inflammatory processes in the kidneys, which potentially results in glomerulosclerosis^28^.

Although IM has been investigated in animals, much of the available toxicological data is the result of either short-term or quite high-dose exposure regimens, but many of them focus on the traditional clinical biochemistry and oxidative stress endpoints^29^. Minor information regarding the combined role of lipid peroxidation, essential pro-inflammatory mediators (such as the NLRP3 inflammasome), and immunohistochemical proliferation phenotypes in both liver and kidney during sub-chronic low exposure through the consumption of drinking water, particularly in commercial IM preparations with co-formulants, is known. This gap might be important in providing a better understanding of the relationship between sub-chronic IM exposure and coordinated oxidative-immune-inflammatory reactions. Moreover, it can show the relationship between these processes and modifications in hepatorenal proliferative markers. Thus, we examined the negative impacts of IM on hepatorenal functions, evaluated oxidative stress biomarkers, proinflammatory mediators (TNF-α, IL −6, and NLRP3 inflammasome), proliferation biomarkers (ASAM, PCNA, and Ki −67), and determined histopathological alterations at a lower dosage level with sub-chronic intake in drinking water.

## Materials and methods

###  Chemicals and reagents

A commercial formulation of IM (35% technical grade w/v in suspended concentrate) was acquired from the Pesticides Analytical Department, Central Agricultural Pesticides Laboratory, manufactured by Hebei Veyong Bio-chemical Co., Ltd., China. Additional analytical-grade chemicals were procured from Sigma Aldrich, USA.

###  Animals

Fifty-five adult male albino rats (*Rattus norvegicus*), weighing 170 ± 10 g, were used in this study (25 for LD_50_ estimation and 30 for sub-chronic study). They were procured from the Animal House of the Mammalian and Aquatic Toxicology Department at the Central Agricultural Pesticides Laboratory, Agricultural Research Centre, Dokki, Giza, Egypt. All animal procedures adhered to the ARRIVE guidelines and Ethical Guidelines established by the Committee of Cairo University for institutional animal care and use (CU/I/F/34/22). Animals were randomly housed in polypropylene cages featuring stainless steel wire lids and clean wood shavings as bedding material, maintained under standard temperature conditions (25 ± 2 °C) with a regular 12-hour light/dark cycle. They were provided unrestricted access to a standard pellet diet and fresh tap water ad libitum.

###  Acute lethal dose study

This study involved twenty-five animals, categorized into five groups, with five rats in each group (5 groups; *n* = 5/group). The acute median lethal dosage (LD_50_) of IM was determined using the methodology established by Weil’s^30^. The initial group administered distilled water orally and designated as the control group. The rats in the treated groups were administered four distinct dosages of IM (555.56, 800, 1152, and 1658.88 mg active ingredient/kg body weight) via oral gavage. The indicators of acute toxicity and the mortality rates in the IM groups were documented. The LD_50_ evaluation was carried out to describe the hazard potential of the tested formulation under the specified experimental settings and to support the safety of dose selection for the sub-chronic trial.

###  Experimental design (sub-chronic study)

The present investigation utilized thirty rats (5 groups; *n* = 6/group). Group (1) administered tap water and served as the control group. Group (2) was administered IM at a concentration of 0.3 ppm. Group (3) was administered IM at a concentration of 3.0 ppm. Group (4) was administered IM at a concentration of 30 ppm. Group (5) was administered IM at a concentration of 60 ppm. The IM doses were determined based on the reference value of the Acceptable Daily Intake (ADI: 0.06 mg/kg), with the lowest dose being five times the ADI^31^. These doses were chosen to ensure survival or, at a minimum, no clinical signs of toxicity, which includes environmental conditions typically considered harmless to humans. All rats received their doses via drinking water. Freshly solutions of IM were made in drinking water at the necessary concentrations during the exposure period. All groups of rats were examined daily for clinical indications and weighed weekly during the sub-chronic trial (90 days).

To assess hepatic and renal responses to recurrent IM ingestion via drinking water, a 90-day exposure duration was chosen as a sub-chronic regimen. As a cautious reference for low-exposure settings, the human acceptable daily intake (ADI; 0.06 mg/kg/day) guided dose selection; nevertheless, we recognize that the ADI is a human health-based benchmark rather than a toxicological threshold specific to rats. To evaluate dose-dependent effects during sub-chronic exposure, the chosen concentrations were meant to cover low to moderate drinking-water levels.

###  Sample collection

Rats underwent overnight fasting; blood samples were collected from the retro-orbital plexus into test tubes and allowed to coagulate for 30 min at room temperature. The serum was then separated using centrifugation at 3600 rpm for 15 min and stored at −20 °C for hepatic and renal function assessments. At the end of the experimental period, the animals were anaesthetized via intraperitoneal injection of sodium pentobarbital (60 mg/kg). Adequate anesthesia was verified by the lack of pedal and corneal responses. Animals were subsequently terminated humanely through cervical dislocation.

Liver and kidney tissue samples were quickly excised, rinsed with ice-cold normal saline to remove blood cells, blotted dry with filter paper, and weighed. The initial portion was homogenized in 1.17% KCl using a Potter-Elvehjem homogenizer, then centrifuged at 10,000 rpm at 4 °C for 20 min. The supernatant was collected and stored at −80 °C for biochemical analysis. The remaining hepatic and renal tissues were fixed in 10% buffered formalin for 24 h, then transferred to 70% ethanol for histopathological and immunohistochemical examination.

### Biochemical assay

The levels of ALT (CAT no. 8.05.22.0.0250), AST (CAT no. 8.05.19.0.0250), ALP (CAT no. 8.05.04.0.0250), TP (CAT no. 8.05.37.0.0250), urea (CAT no. 8.05.41.0.0250), and creatinine (CAT no. 8.05.16.0.0250) were estimated using commercial kits from Atlas Medical, Ludwig-Erhard, Germany.

### Proinflammatory cytokines assay

The hepatic and renal tissue samples were analyzed for inflammatory cytokine (IL-6, TNF-α, and NLRP3) concentrations, as indicators of inflammation, by utilizing enzyme-linked immunosorbent assay kits in rats. The samples were analyzed following the manufacturer’s guidelines (Immunoconceptin, Sacramento, USA).

###  Oxidative stress parameters

The TP concentration in the hepatic and renal samples was estimated calorimetrically using the^32^, with bovine serum albumin as the standard. The levels of MDA, a byproduct of lipid peroxidation, were evaluated in the hepatic and renal samples using the method established by^33^. The concentration of reduced glutathione (GSH) was quantified calorimetrically using Ellman’s reagent^34^. The glutathione-S-transferase (GST) activity was assessed using the established method^35^. The glutathione peroxidase (GPX) activity was estimated using the established protocol^36^. The catalase (CAT) activity was assessed using the method described by^37^.

###  Histopathological and immunohistochemical examination

Fixed hepatic and renal tissue samples from rats across various groups were processed using standard histological methods. This involved dehydration with increasing concentrations of alcohol (80%, 90%, and 100%), followed by embedding in paraffin wax. Five-micrometer-thick sections were cut, and every third section was placed on glass slides. The slides were stained with hematoxylin and eosin using established techniques for histological examination^38^. Using a semiquantitative approach, stained hepatic and renal sections were systematically examined for pathological changes. Changes were measured by frequency across ten randomly chosen fields per section under light microscopy at a magnification of 100x (*n* = 5 specimens per group). The parameters assessed in the liver included hepatocyte degeneration (characterized by vacuolation and swelling), obstructed sinusoids, occluded central and portal veins, mononuclear cell infiltration, pyknotic nuclei, and thickened walls of the portal vein; renal characteristics comprised eosinophilic secretion within the tubular lumen (indicative of protein casts), interstitial mononuclear cell infiltration, fibrotic accumulation, and vascular congestion.

Immunohistochemical evaluation was conducted to identify the ASAM, PCNA, and Ki-67 within 4-µm-thick paraffin-embedded sections derived from liver and kidney tissues, employing traditional avidin-biotin-peroxidase complex techniques. After deparaffinization, the sections underwent antigen retrieval in a Tris-EDTA buffer (pH 9.0) for ASAM and a citrate buffer (pH 6.0) for PCNA and Ki-67. Next, 3% H2O₂ was applied to inhibit endogenous peroxidase activity, and 5–10% bovine serum albumin (BSA) was added to phosphate-buffered saline (PBS) for an hour at room temperature. The primary antibodies, rabbit polyclonal anti-ASAM (16127-1-AP, diluted 1:500), mouse monoclonal anti-PCNA (PC10 or 10205-2-AP, diluted 1:2000), and rabbit polyclonal anti-Ki-67 (ab15580, diluted 1:300), were added and left to incubate overnight at 4 °C. The sections were then treated with biotinylated secondary antibodies (1:500, for one hour), streptavidin-HRP conjugate was administered for thirty minutes, and chromogenic development was carried out using 3,3’-diaminobenzidine (DAB) as a substrate. This produced prominent brown staining that was confined to the nuclei (PCNA, Ki-67) or the cytoplasmic/membranous regions (ASAM). Positive control specimens included regenerating liver tissue for ASAM and PCNA, as well as proliferating renal tubules for Ki-67; conversely, negative controls were processed in the absence of primary antibodies. The slides were subsequently counterstained utilizing Harris hematoxylin, subjected to dehydration, cleared in xylene, and mounted with DPX for subsequent examination via light microscopy.

Immunohistochemical analysis was performed semi-quantitatively using ImageJ software (version 1.54, NIH, USA) to determine the proportion of the area with positive staining. Digital images were obtained at a magnification of 100× using a light microscope with a high-resolution camera. The stained regions (identified by a brown chromogen signifying ASAM cytoplasmic/membranous positivity or nuclear positivity for PCNA/Ki-67) were subject to analysis, and the percentage of the area was calculated using the formula %Area = (positive stained area/total annotated field area) × 100, averaged from 10 randomly selected non-overlapping fields per section (*n* = 5 animals/group). Statistical analysis was performed on the results presented as mean ± standard error. Histopathological and immunohistochemical assessments were carried out on coded slides to minimize the risk of observer bias during scoring and image analysis.

###  Statistical analysis

The statistical analysis was carried out using SPSS software (SPSS for Windows, version 25.0, IBM-SPSS-Inc., USA). The data is shown as the mean (M) ± standard error of the mean (SEM). The probability value (*P* ≤ 0.05) is statistically significant, as assessed by one-way ANOVA and Tukey’s post-hoc multiple comparison among the experimental groups.

## Results

###  Acute oral LD_50_ of IM

The mortality rates for the specified doses were 0/5 at 555.56 mg/kg, 3/5 at 800 mg/kg, 4/5 at 1152.0 mg/kg, and 4/5 at 1658.88 mg/kg. The estimated oral LD_50_ of IM is 837.31 mg/kg body weight.

### Clinical signs, body weight, and tissue weight

All experimental groups of IM-exposed rats displayed no signs of toxicity or mortality during the sub-chronic research. Moreover, the continuous administration of IM at different doses did not produce any significant change in the body weight of the rats, save for a marked decrease noted in those receiving a dosage of 0.3 ppm compared to the control group. No significant change was pointed out in the relative weights of the livers and kidneys of treated animals across all groups, except for the rats receiving 30 ppm, which had a marked increase in the relative weight of the kidneys (Table 1).

**Table 1:** Effect of different doses of IM in drinking water on body weight and relative organ weights of male albino rats for 90 days.

Parameters	Groups				
	Control	IM (0.3 ppm)	IM (3.0 ppm)	IM (30 ppm)	IM (60 ppm)
Body weight (g)	352.6 ± 14.23	300.0 ± 4.18^acde^	349.0 ± 13.17^b^	337.0 ± 11.58^b^	352.6 ± 9.15^b^
Relative liver weight (%)	2.57 ± 0.05	2.7 1 ± 0.09	2.53 ± 0.116	2.61 ± 0.08	2.76 ± 0.07
Relative kidney weight (%)	0.57 ± 0.02	0.59 ± 0.02^d^	0.57 ± 0.018^d^	0.69 ± 0.06^abce^	0.57 ± 0.009^d^

Each value represents the mean ± SE for five animals with a p-value (*P* < 0.05). The letters a, b, c, d, and e denote significant change versus control, 0.3 ppm, 3.0 ppm, 30 ppm, and 60 ppm groups, respectively.

###  Effect of IM on the serum hepatorenal biomarkers

Table 2 presents a significant elevation (*p* < 0.05) in hepatic enzyme activities of ALT (F = 16.44) and AST (F = 9.62) in a dose-dependent manner at 3, 30, and 60 ppm, in comparison to the control group. However, a non-significant elevation in ALP activity was observed at 3.0 ppm; substantial increases were noted at 30 and 60 ppm, in comparison to the control. In contrast to the control group, the top three treatment groups exhibit a modest reduction in total protein content. About renal function metrics, there is a notable elevation in urea (F = 17.899) and creatinine levels (F = 20.398) at 30 and 60 ppm, relative to the control group. In contrast, groups administered IM at 0.3 and 3.0 ppm had no statistically significant elevation in urea and creatinine levels (Table 2).

**Table 2:** Effect of different doses of IM in drinking water on liver and kidney function in male albino rats for 90 days.

Parameters	Groups				
	Control	IM (0.3 ppm)	IM (3.0 ppm)	IM (30 ppm)	IM (60 ppm)
AST (U/L)	95.47 ± 4.98	98.91 ± 3.84^de^	109.56 ± 3.73^ae^	116.78 ± 2.23^ab^	121.96 ± 2.83^abc^
ALT (U/L)	38.73 ± 2.27	40.90 ± 1.85^cde^	48.41 ± 1.44^abe^	52.48 ± 2.05^abe^	59.07 ± 2.51^abcd^
ALP (U/L)	116.30 ± 6.35	109.59 ± 5.68^cde^	128.98 ± 5.35^be^	139.50 ± 5.79^abc^	155.42 ± 5.22^ab^
Total protein (g/dL)	10.55 ± 0.65	10.99 ± 0.58^e^	10.12 ± 0.64	9.77 ± 0.40	9.29 ± 0.35^b^
Urea (mg/dL)	26.94 ± 2.07	27.42 ± 1.73^de^	32.07 ± 1.14^de^	38.75 ± 1.80^abce^	45.74 ± 2.46^abcd^
Creatinine (mg/dL)	0.53 ± 0.03	0.58 ± 0.04^de^	0.6 6 ± 0.04^e^	0.79 ± 0.04^abe^	1.14 ± 0.08^abcd^

Each value represents the mean ± SE for five animals with a p-value (*P* < 0.05). The letters a, b, c, d, and e denote significant change versus control, 0.3 ppm, 3.0 ppm, 30 ppm, and 60 ppm groups, respectively.

###  Effect of IM on proinflammatory cytokine levels

In hepatic tissue, IM significantly raised IL-6 (F = 21.632), TNF-α (F = 19.81), and NLRP3 (F = 3.18) expression levels in a dose-dependent manner, compared to the control group (Table 3). In renal tissue, IM significantly boosted the expression levels of IL-6, TNF-α, and NLRP3 at 30 and 60 ppm, relative to the control group. At dosages of 0.3 and 30 ppm, there was a non-significant elevation in IL-6, TNF-α, and NLRP3 levels, relative to the control group (Table 3).

**Table 3:** Effect of different doses of IM in drinking water on inflammatory biomarkers in the liver and kidney of male albino rats for 90 days.

Parameters		Groups				
		Control	IM (0.3 ppm)	IM (3.0 ppm)	IM (30 ppm)	IM (60 ppm)
Liver	IL-6 (pg/mg protein)	73.73 ± 2.30	73.76 ± 1.66^cde^	87.89 ± 3.75^abe^	94.89 ± 2.77^abe^	106.85 ± 4.10^abcd^
	TNF-α (pg/mg protein)	253.36 ± 11.40	267.56 ± 10.49^de^	281.90 ± 10.72^de^	341.20 ± 12.52^abce^	377.40 ± 13.87^abcd^
	NLRP3 (pg/mg protein)	12.86 ± 1.23	13.66 ± 0.81^e^	14.65 ± 1.45	16.80 ± 0.84^a^	17.54 ± 1.18^ab^
Kidney	IL-6 (pg/mg protein)	38.12 ± 1.73	40.25 ± 2.48	41.86 ± 2.42	43.99 ± 1.89^a^	50.27 ± 2.03^a^
	TNF-α (pg/mg protein)	147.27 ± 4.79	154.49 ± 3.33^de^	160.92 ± 3.25^de^	181.52 ± 8.96^abc^	197.81 ± 6.72^abc^
	NLRP3 (pg/mg protein)	9.57 ± 0.94	10.27 ± 0.79^e^	10.97 ± 0.37^e^	11.86 ± 0.81^ae^	14.70 ± 0.64^abcd^

Each value represents the mean ± SE for five animals with a p-value (*P* < 0.05). The letters a, b, c, d, and e denote significant change versus control, 0.3 ppm, 3.0 ppm, 30 ppm, and 60 ppm groups, respectively.

### Effect of IM on hepatorenal redox status

In comparison to the control group, there was a statistically significant (*p* < 0.05) elevation in hepatic MDA levels (F = 4.325) across all tested groups (0.3, 3, 30, and 60 ppm). Nonetheless, the decrease in GSH and GPX activities was non-significant across all groups in hepatic tissue. The GST activity (F = 2.486) was significantly reduced in all treated groups in hepatic tissue compared to the control group. Furthermore, hepatic CAT activity (F = 11.735) significantly diminished at 30 ppm and 60 ppm (Table 4).

**Table 4:** Effect of different doses of IM in drinking water on liver and kidney redox status in male albino rats for 90 days.

Parameters		Groups				
		Control	IM (0.3 ppm)	IM (3.0 ppm)	IM (30 ppm)	IM (60 ppm)
Liver	MDA (nmol/g)	276.84 ± 19.79	400.55 ± 34.39^a^	399.60 ± 35.33^a^	414.0 ± 35.51^a^	388.74 ± 9.11^a^
	GSH (µmol/g)	11.71 ± 0.13	11.99 ± 0.65	11.27 ± 0.56	11.47 ± 1.20	10.69 ± 1.16
	GST (U/mg protein)	26.91 ± 1.02	24.34 ± 0.92^a^	23.43 ± 1.00^a^	24.43 ± 0.63^a^	24.34 ± 0.36^a^
	GPX (U/mg protein)	20.31 ± 0.74	21.15 ± 0.54	21.16 ± 1.36	21.40 ± 0.31	21.81 ± 0.47
	CAT (U/mg protein)	911.66 ± 59.68	750.77 ± 55.96^ce^	965.78 ± 27.60^bde^	667.75 ± 64.17^ace^	1155.43 ± 62.59^abcd^
Kidney	MDA (nmol/g)	404.88 ± 13.47	469.72 ± 39.90	516.04 ± 29.17^a^	470.58 ± 23.78	498.61 ± 21.49^a^
	GSH (µmol/g)	11.25 ± 0.67	9.59 ± 0.73^d^	10.32 ± 0.84^d^	6.60 ± 0.44^abce^	11.01 ± 0.45^d^
	GST (U/mg protein)	3.77 ± 0.14	1.72 ± 0.16^a^	1.84 ± 0.14^ad^	1.31 ± 0.12^ace^	2.07 ± 0.23^ad^
	GPX (U/mg protein)	22.35 ± 0.32	18.90 ± 0.47^ace^	21.05 ± 0.46^b^	19.05 ± 1.12^a^	21.08 ± 0.80^b^
	CAT (U/mg protein)	1034 ± 24.07	953.15 ± 14.48^e^	954.60 ± 34.52^e^	963.54 ± 62.79^e^	1128.88 ± 49.11^bcd^

Each value represents the mean ± SE for five animals with a p-value (*P* < 0.05). The letters a, b, c, d, and e denote significant change versus control, 0.3 ppm, 3.0 ppm, 30 ppm, and 60 ppm groups, respectively.

Compared to the control group, the groups given IM at 3.0 and 60 ppm showed significant increases in kidney MDA levels (F = 2.45), but the increases in the 0.3 and 30 ppm groups were not statistically significant. However, renal GST activity (F = 32.20) decreased significantly in all IM-treated groups. Additionally, renal GPX activity (F = 4.451) was significantly reduced in the 3.0 and 30 ppm groups, while the other groups showed nonsignificant reductions. Moreover, GSH concentration (F = 8.352) decreased markedly in rats exposed to 30 ppm IM, although CAT (F = 3.474) showed no significant change (Table 4).

### Histopathological observations

**Fig. 1:**
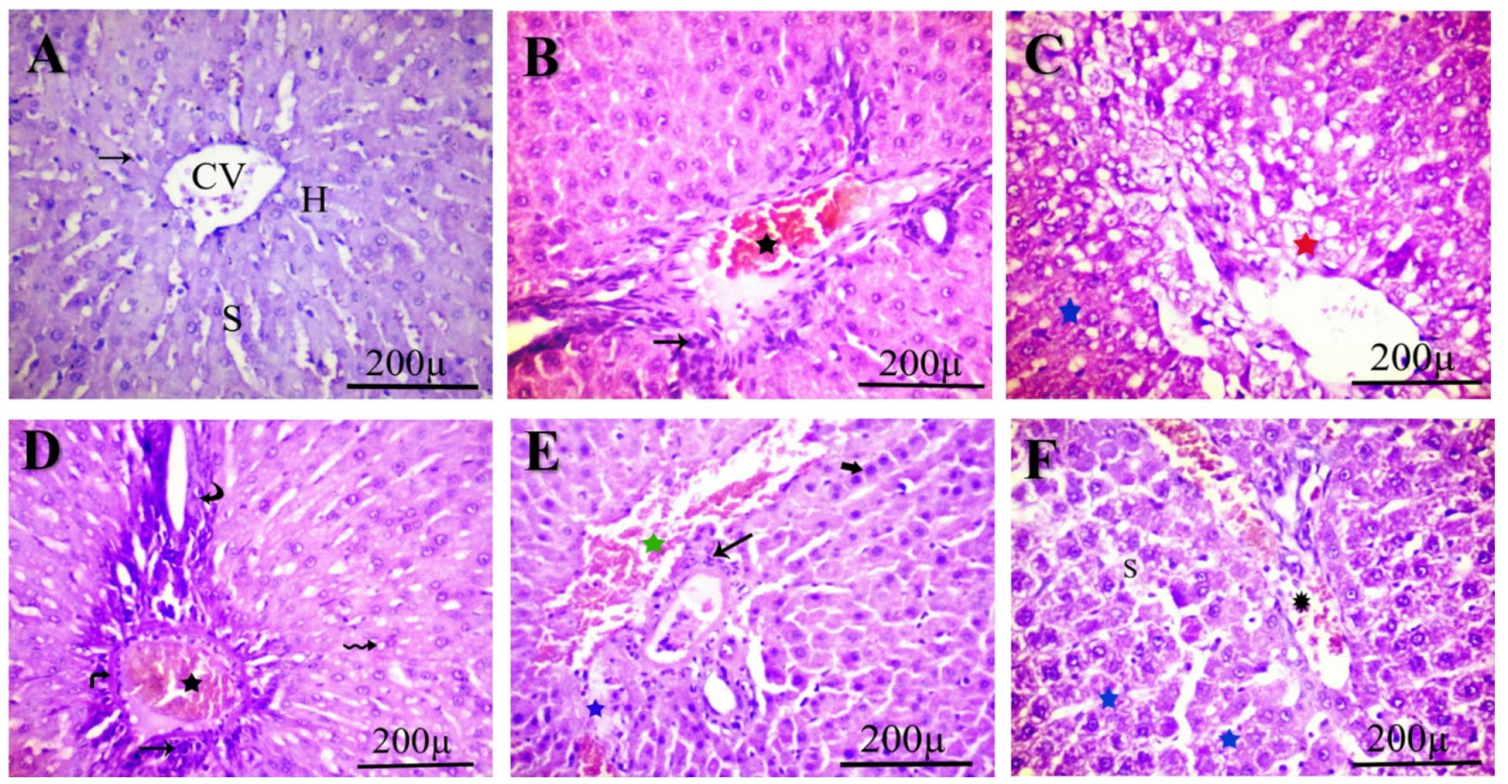
Photomicrographs of hematoxylin-eosin-stained sections from all studied groups show normal hepatic morphology in the control group (**A**), central vein (CV), hepatic cell (H), sinusoid (S), and Kupffer cell (arrow). IM-treated groups at 0.3 ppm (**B**) show lymphatic infiltration and congested portal veins. At 3.0 ppm (**C** &** D**), the presence of steatosis and necrotic areas, severe congestion, and vacuolation is shown. At 30 ppm (**E**), they show pyknotic hepatocytes, together with necrotic dilated blood sinusoids. At 60 ppm (**F**), show necrosis and congestion. Lymphatic infiltration (arrow), congested portal vein (black star), steatosis (red star), cytoplasmic vacuolation (wavy arrow), bile duct proliferation (curved arrow), dilated congested sinusoid (green star), necrotic change (blue star), pyknosis (bold arrow), and thick wall (turn arrow). Scale bar 200 μm.

Liver sections from the control rats (Fig. 1-A) exhibit a normal histological architecture, characterized by an intact central vein and endothelial lining. Conversely, the group administered IM at 0.3 ppm (Fig. 1-B) exhibited lymphatic infiltration, disrupted endothelial cells next to the central vein, a congested portal vein, and steatosis. Furthermore, specimens from the IM-treated group at 3.0 ppm (Fig. 1-C & D) exhibited diffuse hepatocellular cytoplasmic vacuolation, necrotic regions, steatosis, rupture of endothelial cells along the central vein, thickening of the portal vein wall, lymphatic infiltration, and proliferation of bile ducts. The group treated with 30 ppm of IM (Fig. 1-E) had substantial degradation of normal structure, characterized by an increase in pyknotic cells, notable congestion, and steatosis. The wall of the hepatic portal vein thickened and became congested, while the endothelial cells of the central vein were compromised, and lymphatic tissue proliferated surrounding the portal region alongside an increase in bile ducts. In the 60 ppm IM group (Fig. 1-F), histological alterations comprised disorganized and degraded hepatocytes, necrosis, steatosis, enlarged sinusoids, and a distended, dilated, and congested central vein, in addition to congestion of the hepatic portal vein and lymphatic infiltration.

**Fig. 2:**
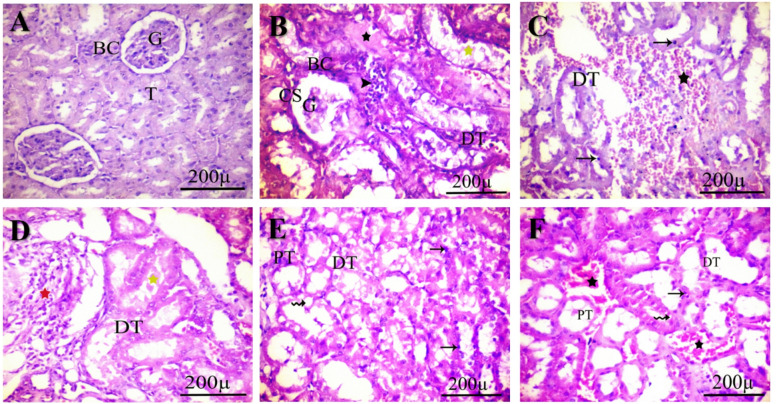
Photomicrographs of hematoxylin-eosin-stained sections from the control group (**A**) show normal renal morphology of Bowman’s capsule (BC), renal tubules (T), and glomeruli (G). The IM-treated group 0.3 ppm (**B**) shows degenerated tubules, atrophy of renal corpuscles, and areas of hyaline casts and mononuclear cell aggregation between tubules. At a dosage of 3.0 ppm (**C**), severe congestion, pyknotic dark nuclei, and deteriorated tubules were observed. At 30 ppm (**D** &** E**), they exhibited widening of the tubular lumen with precipitation of hyaline casts and inflammatory cell invasion, pyknosis, and vacuolation. At 60 ppm (**F**), show pyknotic nuclei, cytoplasmic vacuoles, intratubular congestion, and widening of the tubular lumen proximal tubule (PT) and distal tubule (DT). Lymphatic infiltration (arrowhead, red star), degenerated tubule (DT), pyknotic dark nuclei (arrow), congestion (*), hyaline casts (yellow star), cytoplasmic vacuoles (wavy arrow), and wide capsular space (CS). Scale bar 200 μm.

Microscopic analysis of renal tissue slices from control rats reveals a normal histological architecture of Bowman’s capsule, renal tubules, and glomeruli (Fig. 2-A). Conversely, the group administered 0.3 ppm of IM showed reduced kidney architecture and compromised distal tubules. Mononuclear cell aggregates emerged between the tubules, accompanied by cytoplasmic vacuolation. Regions exhibiting hyaline casts and atrophied glomeruli with larger capsular gaps were noted (Fig. 2-B). The cohort subjected to 3.0 ppm of IM exhibited reduced glomerular size, increased interstitial spaces, and compromised epithelial cells in both proximal and distal tubules. The interstitial tissue between the tubules exhibited notable edema, hemorrhage, hyaline cast formation, and elevation in lymphocyte count (Fig. 2-C). The group administered 30 ppm (Fig. 2-D & E) demonstrated significant renal alterations, characterized by reduced glomerular size with enlarged interstitial spaces, cellular swelling, inflammation, congestion, and necrotic tissue in both proximal and distal tubules, as well as disrupted epithelial cells and hyaline casts in the distal tubules. In the kidney segment from the 60 ppm group, distinct structural alterations were seen, including enlarged glomeruli with vacuoles, expanded tubular gaps, and the deposition of hyaline casts. The periglomerular areas exhibited infiltration of inflammatory cells and congestion.

### Histomorphology analysis

The hepatocyte degeneration in the IM groups at doses of 0.3, 30, and 60 ppm showed a non-significant increase (*p* > 0.05), compared to the control group. The obstructed sinusoid in the 3.0 and 30 ppm treatment groups increased by 11.11% and 55.56%, respectively, relative to the control group (*p* > 0.05). The clogged veins increased substantially in the three higher groups (3, 30, and 60 ppm) by 60%, 90%, and 70%, respectively, compared to the control group. Mononuclear cell infiltration rose by 30%, 60%, 40%, and 40% in the IM-treated groups (0.3, 3, 30, and 60 ppm), respectively. Pyknotic nuclei showed similar results at 40%, 120%, 230%, and 100%, respectively, compared to the control group. Fatty alteration (50%, 60%, and 50%) and thickened portal vein walls (50%, 70%, and 10%) showed significant increases at the three higher doses of IM (3, 30, and 60 ppm), respectively, relative to the control group.

Concerning the kidneys, there was an escalation in tubular epithelial cell degeneration (66.67%, 100%, 255.56%, and 155.6%), eosinophilic secretion within the tubular lumen (240%, 120%, 400%, and 540%), and interstitial mononuclear cell infiltration (40%, 10%, 60%, and 140%) in all IM-treated cohorts relative to the control group. Fiber accumulation exhibited a notable increase of 40% at the 30 ppm dosage level. The congestion within the tubules rose significantly by 200%, 171.43%, 257.14%, and 200% in the IM-treated groups compared to the control. The quantity of decreased glomeruli rose markedly at 0.3 ppm (100%) and 30 ppm (180%) concentrations, whereas glomerular enlargement was observed at 3.0 ppm (60%).

### Immunohistochemical analysis

**Fig. 3:**
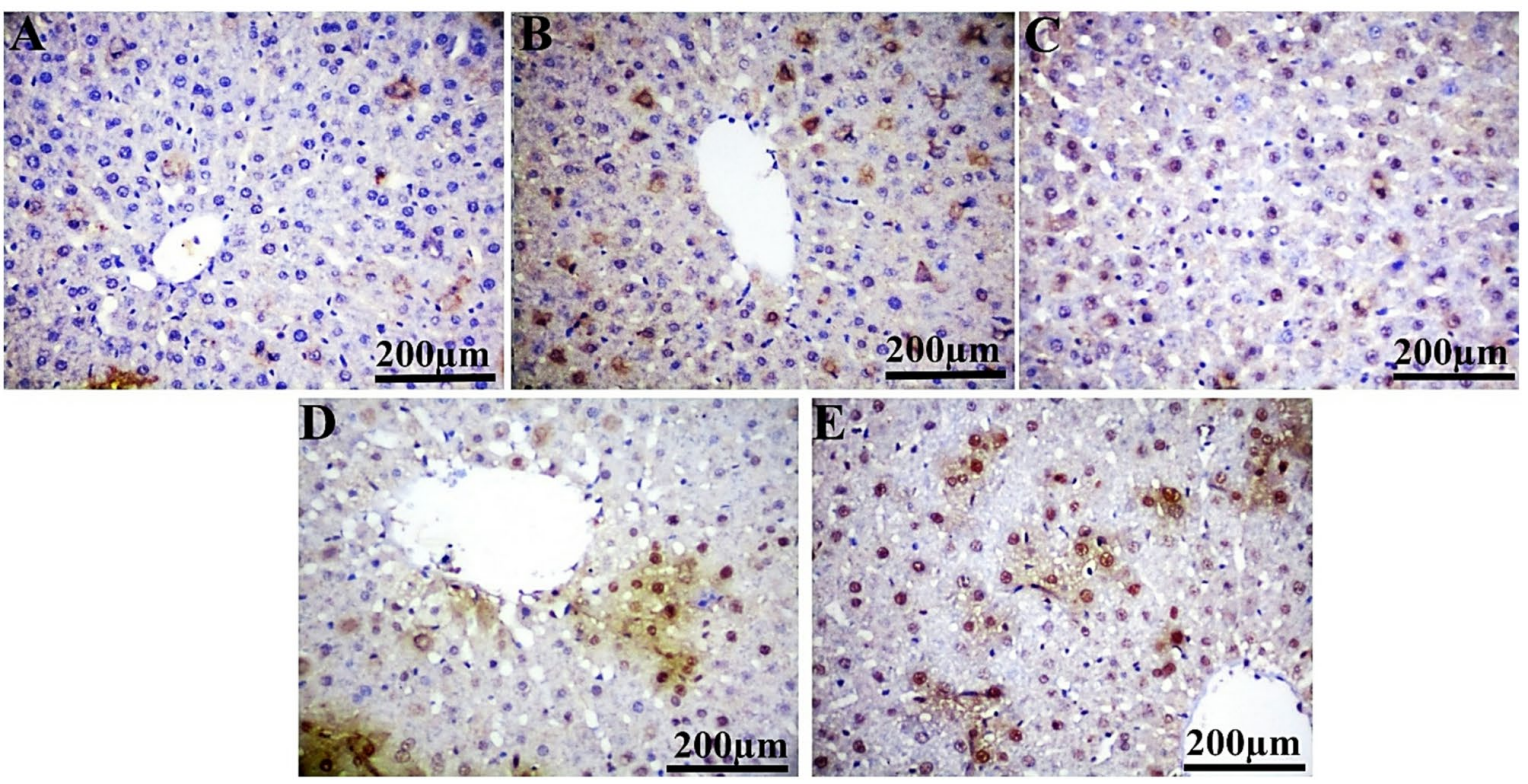
Photomicrographs of immunohistochemically stained hepatic sections for PCNA protein expression of the control (**A**), IM groups at doses 0.3 ppm (**B**), 3 ppm (**C**), 30 ppm (**D**), and 60 ppm (**E**). Scale bar 200 μm.

**Fig. 4:**
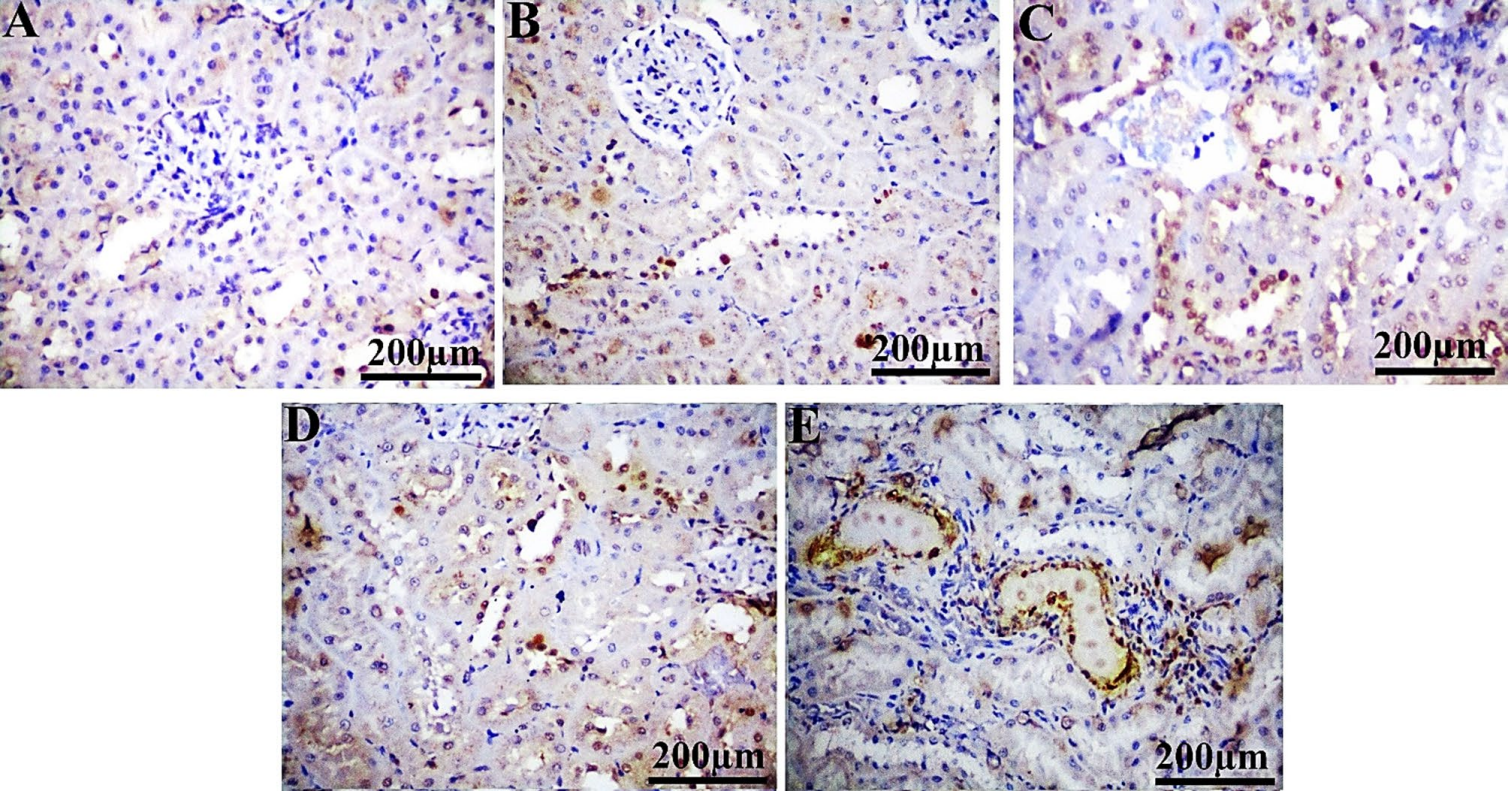
Photomicrographs of immunohistochemically stained renal sections for PCNA protein expression of the control (**A**), IM groups at doses 0.3 ppm (**B**), 3 ppm (**C**), 30 ppm (**D**), and 60 ppm (**E**). Scale bar 200 μm.

**Fig. 5:**
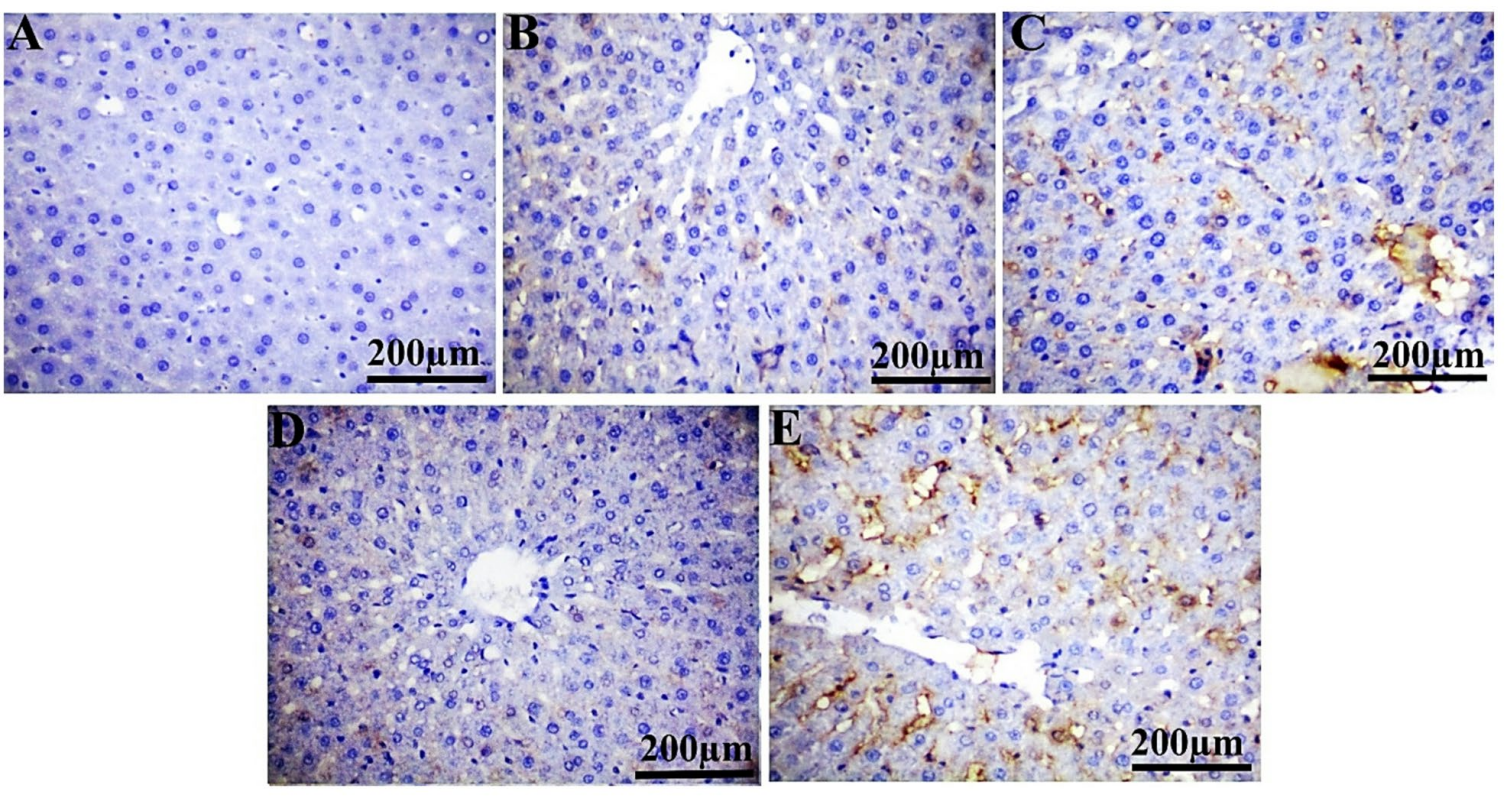
Photomicrographs of immunohistochemically stained hepatic sections for Ki67 protein expression of the control (**A**), IM groups at doses 0.3 ppm (**B**), 3 ppm (**C**), 30 ppm (**D**), and 60 ppm (**E**). Scale bar 200 μm.

**Fig. 6:**
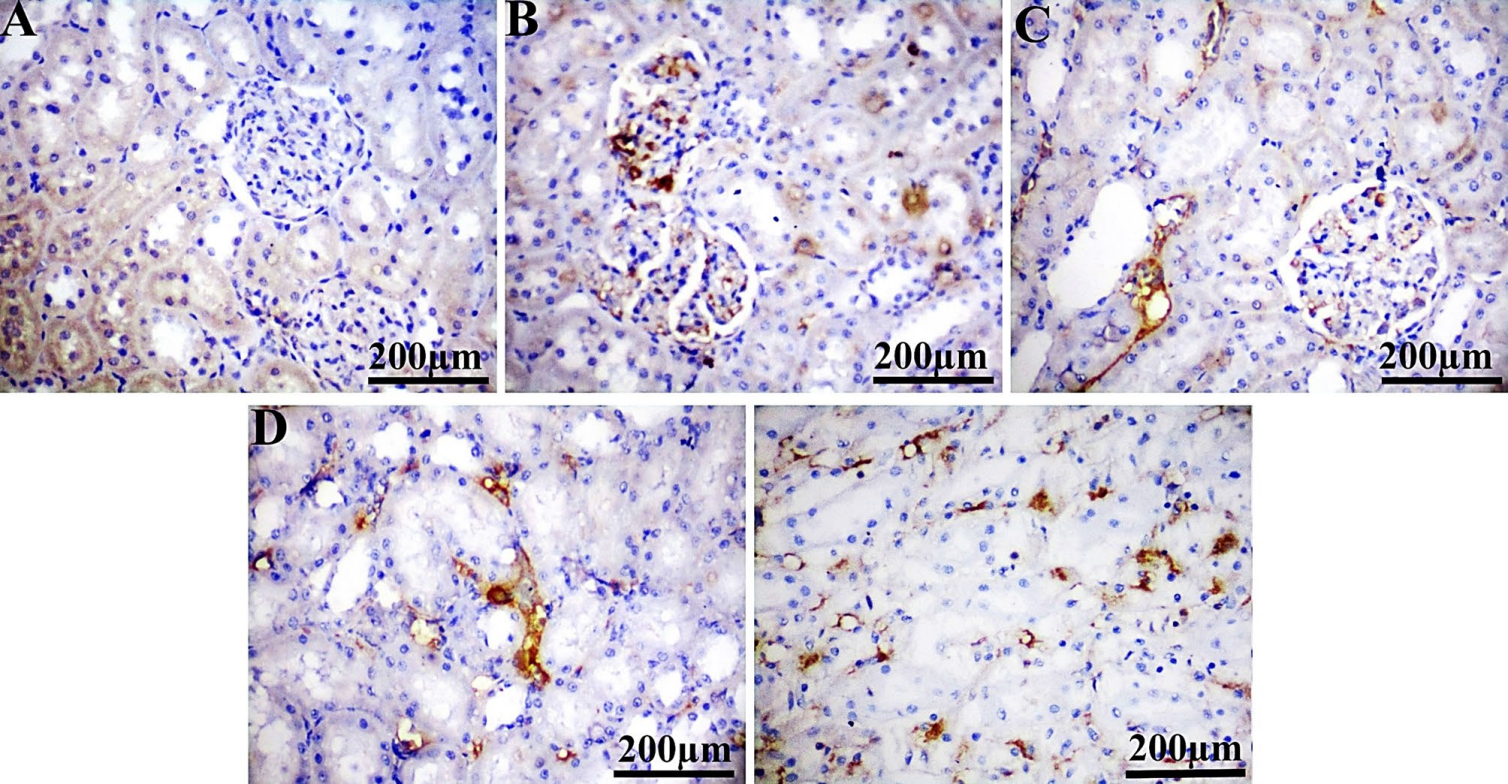
Photomicrographs of immunohistochemically stained renal sections for Ki67 protein expression of the control (**A**), IM groups at doses 0.3 ppm (**B**), 3 ppm (**C**), 30 ppm (**D**), and 60 ppm (**E**). Scale bar 200 μm.

 In comparison to the control group, PCNA showed a substantial positive expression in liver (29.92%, 20.19%, 14.77%, and 35.23%) and kidney (33.78%, 28.62%, 54.65%, and 74.50%) sections from IM-treated groups at dosage levels of 0.3, 3, 30, and 60 ppm, respectively (Table 5; Figs. 3 and 4). Notably, Ki-67 expression is significantly reduced at 0.3 ppm (9.23%), with no significant variation observed at 3, 30, and 60 ppm in the liver and kidney (all doses) of rats administered IM, compared to the control group (Table 5; Figs. 5 and 6). The ASAM protein demonstrated non-significant changes in both the liver and kidney in all IM-treated groups relative to the control (Table 5; Figs. 7 and 8).

**Table 5:** Effect of different doses of IM in drinking water on the ASAM, PCNA, and Ki-67 immunoreactivity in the liver and kidney of male albino rats for 90 days.

Parameters		Groups				
		Control	IM (0.3 ppm)	IM (3.0 ppm)	IM (30 ppm)	IM (60 ppm)
Liver	PCNA	0.290 ± 0.012	0.377 ± 0.016^ad^	0.348 ± 0.006^a^	0.333 ± 0.007^ab^	0.392 ± 0.007^acd^
	Ki-67	0.308 ± 0.008	0.280 ± 0.010^acde^	0.320 ± 0.005^b^	0.302 ± 0.007^b^	0.321 ± 0.006^b^
	ASAM	0.393 ± 0.010	0.363 ± 0.013	0.384 ± 0.010	0.366 ± 0.008^e^	0.397 ± 0.012^bd^
Kidney	PCNA	0.246 ± 0.010	0.330 ±0.010^ade^	0.317 ± 0.007^ade^	0.381 ± 0.011^abce^	0.430 ± 0.007^abcd^
	Ki-67	0.344 ± 0.008	0.332 ± 0.006^de^	0.326 ± 0.007^ad^	0.302 ± 0.004^abc^	0.312 ± 0.005^ab^
	ASAM	0.384 ± 0.016	0.419 ± 0.025	0.398 ± 0.013	0.400 ± 0.011	0.410 ± 0.012

Each value represents the mean ± SE for five animals with a p-value (*P* < 0.05). The letters a, b, c, d, and e denote significant change versus control, 0.3 ppm, 3.0 ppm, 30 ppm, and 60 ppm groups, respectively.

## Discussion

The widespread use of pesticides in agriculture worldwide to boost plant productivity has resulted in environmental pollution, negatively impacting non-target species, which leads to oxidative and mitochondrial dysfunction^1^. Subsequently, we examined the impact of sub-chronic exposure to low doses of IM in drinking water on liver and kidney health, including oxidative stress levels, and inflammatory markers (TNF-α, IL-6, and NLRP3), and cellular proliferation markers (ASAM, PCNA, and Ki-67).

The estimated oral LD_50_ of IM is 837.31 mg/kg of body weight, indicating the dose required to kill 50% of the animals (rats). Previous research has shown similar LD_50_ values for IM in rats. For example, Kapoor^39^ reported an oral LD50 of 475 mg/kg in rats, while Chandran^40^ found an LD_50_ of 450 mg/kg. Because we employed a formulated product with IM as an active ingredient together with proprietary co-formulants such as solvents, surfactants, or stabilizing agents, the LD50 value can differ^39^.

The lack of clinical symptoms of toxicity or fatality in our study aligns with findings from prior research that reported no significant adverse effects at lower dosages of IM. This finding implied that while the IM would be relatively safe at lower dosages, higher ones might have negative consequences^39^. Furthermore, even though rats had unlimited access to food, the previous study showed a significant decrease in body weight after being exposed to IM, confirming the detrimental effects of pesticides on animal tissues that may hinder nutritional digestion^18^. The decrease in body weight at 0.3 ppm may result from subclinical effects not observable in higher dose groups. As a result, it implies a non-linear dose-response relationship, which necessitates further investigation to understand the underlying mechanics. Furthermore, except for rats given 30 ppm, the slight variations in the relative weights of the liver and kidneys in most treatment groups are consistent with results from previous research^39^. Moreover, the rise in kidney weight at 30 ppm points to possible nephrotoxicity at greater concentrations^18^.

Our study showed a dose-dependent increase in ALT and AST activities, along with elevated ALP activity at doses of 30 and 60 ppm after 90 days of exposure to IM. These results support other studies that reported increases in ALT, AST, and ALP activities following IM exposure through drinking water^29^. The rise in these enzymes may be caused by disrupted production, along with changes in liver membrane permeability^41^, leading to liver injury and hepatocyte necrosis^18^. The compromised integrity of cell membranes from hepatocyte degeneration may cause transaminases to leak into the bloodstream. As a result, liver cell injury could decrease albumin production^42^, which in turn lowers total protein levels after IM intoxication, as observed in our study and noted by^18^.

The current study’s data showed that urea and creatinine levels had significantly increased, particularly in the two higher IM-treated groups. Higher levels of kidney function markers indicated that IM exposure may have nephrotoxic effects^43^, according to earlier study. Increased tissue or dietary protein breakdown and/or delayed renal nitrogen waste clearance might result in elevated creatinine levels^44^. These recommendations might be connected to rates of protein catabolism, which could subsequently impair the kidneys’ capacity to eliminate urea and creatinine^45^.

Notably, during the 90-day exposure period, rats treated with IM exhibited biochemical and histological changes associated with hepatorenal stress. The oxidative stress data show that lipid peroxidation (measured by MDA) increased in the liver at all tested concentrations and in the kidney at specific concentrations. Whereas changes in enzymatic antioxidant defenses and GSH varied, depending on the endpoint and tissue examined. In this respect, substantial increases in hepatic MDA levels may result from a difference between antioxidant capacity and the overproduction of free radicals after exposure to neonicotinoids^46^, as indicated by IM. Additionally, the notable decrease in GSH levels at higher doses suggests a depletion of this essential antioxidant, which may increase hepatic vulnerability to oxidative damage because GSH acts as a substrate for detoxifying enzymes by facilitating GSH-dependent conjugation reactions^47^.

Our data showed non-significant increases in MDA levels (in renal tissue) at lower doses of IM; however, substantial increases were seen at higher doses, indicating that these dosages lead to increased oxidative stress in the kidney^46^. The significant drop in GST and GPX activities seen in all treatment groups suggests reduced antioxidant defenses in the kidney. Moreover, changes in CAT activity suggest that IM exposure may have a minimal effect on this enzyme in renal tissue^48^. Although GSH levels and GST, GPX, and CAT activities decreased significantly in liver tissues, there was no discernible change in renal tissue, except in rats treated with 30 ppm. The absence of significant increases in GSH and GPX levels suggests that, despite increased lipid peroxidation, antioxidant capability may remain unaltered^47^.

The decreased GST activity in kidney tissue may result from detoxification responses that protect cells from oxidative damage by neutralizing lipid peroxidation products, thereby indirectly promoting DNA repair^49^, and by catalyzing the conjugation of electrophilic compounds to thiol groups^50^. Overall, the complex effects on CAT activity, showing reductions at certain doses and increases at others, suggest that the antioxidant response to IM treatment is both dose-dependent and tissue-specific^18^. Therefore, IM can induce oxidative stress and inflammation in a dose-dependent manner, which can lead to DNA damage, protein degradation, lipid peroxidation, and tissue injury, especially in the liver (a detoxification organ) and kidneys (an excretory organ)^51^.

In this investigation, the liver and kidney responded differently to oxidative stress, which could imply tissue-specific variance in sensitivity and compensatory abilities. The absence of statistically significant changes in some enzymatic antioxidant markers at specific exposure levels may indicate compensatory mechanisms or temporal variations in antioxidant responses rather than a lack of oxidative challenge, even though MDA elevations indicate increased lipid peroxidation^50^.

Remarkably, histopathological results showed a clear dose-dependent toxicity of IM on the liver^52^ and kidneys^53^. Prior work revealed that moderate pathological changes were observed in the liver^54^ and kidney^55^ of female rats exposed to higher doses of IM. These findings indicate that IM can induce oxidative stress and inflammation in the liver, causing cumulative damage as the dose increases^18^. Additionally, the observed histological changes, including glomerular atrophy and tubular degeneration, emphasize the nephrotoxic effects of IM^56^. The results highlight the potential risks of IM exposure on liver and kidney health, underscoring the need for further research to fully understand the underlying mechanisms and develop ways to reduce its harmful effects. Consequently, the widespread use of IM emphasizes the importance of stricter regulations and the development of safer alternatives to lessen its impact on non-target organisms.

Although it is believed that neonicotinoids have low toxicity because of their weak interaction with nicotinic receptors, ingesting large amounts can cause severe poisoning^57^. It has been shown that IM causes liver cell damage by damaging the hepatic membrane structure, disrupting defense barriers, and increasing proinflammatory cytokines^58^. This poisoning may be related to the prolonged absorption or elimination of high doses of IM^59^. The current study demonstrated that IM significantly increased the expression levels of proinflammatory cytokines IL-6, TNF-α, and NLRP3 in liver tissue in a dose-dependent way. Our findings are consistent with a previous study showing that IM can cause inflammation and oxidative stress^60^. This effect might result from the overproduction of pro-inflammatory cytokines, including TNF-α, which are linked to inflammatory liver conditions such as fatty liver disease^29^ due to IM exposure. This suggests that IM administration could trigger an inflammatory response in liver tissue, potentially causing liver injury, and explains the severe effects on liver tissues at higher doses^58^.

Particularly at the two higher doses, IM markedly elevated the levels of TNF-α, NLRP3, and IL-6 in renal tissue. This increase suggests that renal tissue is experiencing inflammation, which could result in kidney damage. In this regard, elevated NF-κB expression characterizes the inflammatory response after IM exposure, which in turn increases the release of pro-inflammatory cytokines like TNF-α and IL-1. The breakdown of cytoskeletal proteins and the lipid peroxidation of membrane phospholipids enhance oxidative stress-induced cellular damage, which leads to this action. These oxidative insults may activate pro-inflammatory signaling pathways to initiate a cascade of inflammatory events^61^. This data suggests that IM can have nephrotoxic effects associated with the production of pro-inflammatory cytokines, which are elevated by oxidative stress^62^. Therefore, the dose-dependent nature of these results suggests that exposure to IM marks more significant inflammatory responses, resulting in greater tissue damage. While renal functional impairment became more noticeable at higher exposure levels, in accordance with its excretory function and tubular susceptibility, the liver showed earlier changes in enzymatic activity and more prominent histopathological lesions, consistent with its central role in xenobiotic metabolism.

Remarkably, the immunohistochemical examination of liver and kidney tissues from rats exposed to IM demonstrates notable alterations in the expression of PCNA, Ki-67, and ASAM proteins relative to the control group. In this issue, the elevation of PCNA expression indicates increased cell proliferation in liver than kidney tissues after IM exposure. This may represent a compensatory reaction to tissue injury, as cells attempt to replace those lost due to the toxic effects of IM. This suggests that xenobiotics encourage DNA damage, and the increased PCNA expression detected could be associated with DNA repair^63^.

The absence of Ki-67 expression in the liver at the lowest dosage suggests that cellular proliferation may be suppressed. Moreover, the lack of significant changes at higher concentrations implies that the proliferative response might be more complex and not solely reliant on Ki-67 expression in the liver. Consequently, the marked reduction in Ki-67 expression in the kidneys at high dosages suggests that IM may prevent cell division, potentially resulting in renal damage^64^. While Ki-67 is a sign of active cell-cycle progression, upregulation of PCNA may imply enhanced DNA repair activity and compensating regenerative responses. Therefore, under situations of toxic stress, where activation of repair mechanisms is not always followed by a comparable increase in proliferative activity^65^, divergent expression patterns between PCNA and Ki-67 may emerge^64^. Accordingly, the primary finding is that pesticides can prevent cell division. Since the Ki-67 protein indicates cell division, lower levels of Ki-67 indicate fewer cells dividing. Furthermore, in all IM-treated groups, the immunohistochemistry expression of ASAM protein showed little alterations in the kidney and liver^66^. Moreover, ASAM expression did not show significant changes under the current exposure settings, which may imply that this marker is not significantly modified by the sub-chronic IM regimen examined, or that its responsiveness may be reliant on alternative exposure intensity or duration. This finding emphasizes how crucial it is to assess the toxicological consequences of IM using a variety of indicators.

### Limitations

There are concerns with this study. The first study used only adult male albino rats, which may limit extrapolation to females or other species. However, one significant limitation of the study is the lack of internal dosage measures (e.g., plasma or tissue concentrations of IM), which prevents direct evaluation of bioaccumulation and clearance dynamics in hepatic and renal tissues. Moreover, this study’s inability to assess drinking water intake made it impossible to estimate IM exposure on a mg/kg body weight/day basis. When analyzing the observed dose-response patterns and comparing the outcomes with regulatory or guideline reference values, this factor should be considered.

## Conclusions

The present study indicates that sub-chronic exposure to IM, even at lower doses, is associated with alterations in hepatorenal function in male albino rats. The observed changes are associated with increased oxidative stress, heightened production of inflammatory cytokines, and alterations in markers of cell proliferation. Nonetheless, further research is needed to elucidate the causal mechanisms. Additional research utilizing various dosages, exposure durations, and experimental models is essential for a thorough evaluation of the long-term effects of IM.

## Data Availability

The datasets used and/or analyzed during the current study are available from the corresponding author upon reasonable request.
